# Evaluating Network Readiness for mHealth Interventions Using the Beacon Mobile Phone App: Application Development and Validation Study

**DOI:** 10.2196/18413

**Published:** 2020-07-28

**Authors:** Thomas Foster Scherr, Carson Paige Moore, Philip Thuma, David Wilson Wright

**Affiliations:** 1 Department of Chemistry Vanderbilt University Nashville, TN United States; 2 Macha Research Trust Choma Zambia

**Keywords:** mHealth, network readiness, network assessment, mobile network

## Abstract

**Background:**

Mobile health (mHealth) interventions have the potential to transform the global health care landscape. The processing power of mobile devices continues to increase, and growth of mobile phone use has been observed worldwide. Uncertainty remains among key stakeholders and decision makers as to whether global health interventions can successfully tap into this trend. However, when correctly implemented, mHealth can reduce geographic, financial, and social barriers to quality health care.

**Objective:**

The aim of this study was to design and test Beacon, a mobile phone–based tool for evaluating mHealth readiness in global health interventions. Here, we present the results of an application validation study designed to understand the mobile network landscape in and around Macha, Zambia, in 2019.

**Methods:**

Beacon was developed as an automated mobile phone app that continually collects spatiotemporal data and measures indicators of network performance. Beacon was used in and around Macha, Zambia, in 2019. Results were collected, even in the absence of network connectivity, and asynchronously uploaded to a database for further analysis.

**Results:**

Beacon was used to evaluate three mobile phone networks around Macha. Carriers A and B completed 6820/7034 (97.0%) and 6701/7034 (95.3%) downloads and 1349/1608 (83.9%) and 1431/1608 (89.0%) uploads, respectively, while Carrier C completed only 62/1373 (4.5%) file downloads and 0/1373 (0.0%) file uploads. File downloads generally occurred within 4 to 12 seconds, and their maximum download speeds occurred between 2 AM and 5 AM. A decrease in network performance, demonstrated by increases in upload and download durations, was observed beginning at 5 PM and continued throughout the evening.

**Conclusions:**

Beacon was able to compare the performance of different cellular networks, show times of day when cellular networks experience heavy loads and slow down, and identify geographic “dead zones” with limited or no cellular service. Beacon is a ready-to-use tool that could be used by organizations that are considering implementing mHealth interventions in low- and middle-income countries but are questioning the feasibility of the interventions, including infrastructure and cost. It could also be used by organizations that are looking to optimize the delivery of an existing mHealth intervention with improved logistics management.

## Introduction

A technological revolution is rapidly changing the way health care is approached worldwide. Previously insurmountable logistical challenges such as supply chain management [1], cold reagent storage [2,3], and disease surveillance [4] are being solved by a continuous stream of innovations [5,6]. These solutions, which can range from simple SMS text message appointment reminders [7,8] to more sophisticated approaches such as mobile phone–based clinical-grade electrocardiograms [9], have broad uses and can be tailored to address many scenarios and user groups [5,6,10]. This new class of health care interventions, called mobile health (mHealth), takes advantage of the prevalence of network-connected mobile devices and mobile apps and is being adapted to meet challenges present in low- and middle-income countries to improve health outcomes [11]. With 5.3 billion mobile subscribers worldwide and 90% of the global population covered by wireless signals, a niche has developed for the use of mobile phones in public health campaigns [12-14]. mHealth approaches are growing in popularity in high-income countries, where they provide patients with improved convenience and enable providers to engage their patients in near real-time [15-17]. However, mHealth approaches are also being used in global health settings, where they are implemented to decrease costs, minimize barriers to facilitating care, and provide more useful surveillance data [18-20].

**Figure 1:**
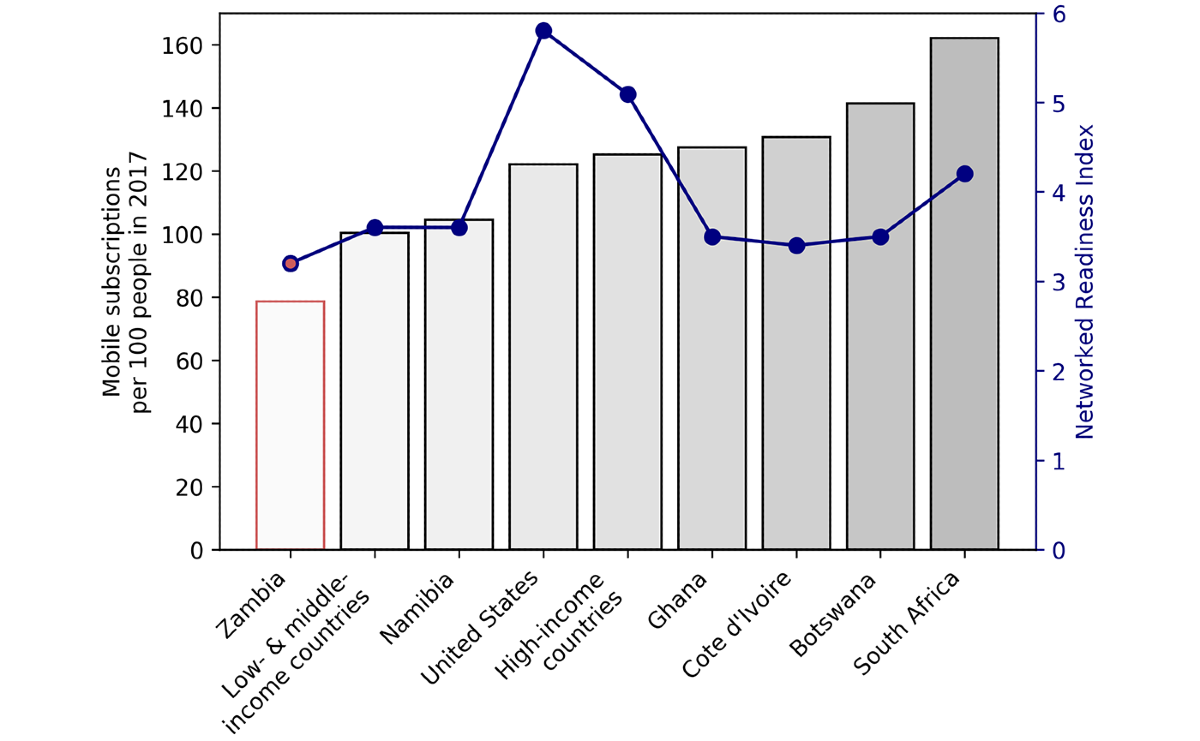
The number of mobile subscriptions per 100 people in 2017 and the World Economic Forum Networked Readiness Index (NRI) for several African nations, the United States, World Bank low- to middle-income countries, and World Bank high-income countries. Data for Zambia, the country where this study was performed, are emphasized in red. The NRI data for low- to middle-income countries and high-income countries are averages of the available NRIs for World Bank countries in each classification (several countries in each group do not have a listed NRI).

The growth of global mHealth has naturally followed the surge in mobile phone uptake. The ubiquity of mobile devices in low- and middle-income countries increased significantly between 2000 and 2015, and it is expected to expand further [21]. In Africa specifically, the average number of mobile phone subscriptions per 100 people increased from 3 to 85 over that same period [21]. In some low- and middle-income countries, the number of mobile subscriptions per person is higher than in many developed countries, including the United States (Figure 1).

In Zambia, where we conducted our study, the World Bank reported a prevalence of 78.6 mobile phone subscriptions per 100 people. Despite the availability of mobile phones in Zambia, the country has a Networked Readiness Index (NRI) value of 3.2/6.0, ranking 116th worldwide. The NRI is a measurement that quantifies a nation’s use of technology to increase international competitiveness. Zambia has approximately one-third fewer mobile phones per capita than the United States and a conspicuously lower NRI score; therefore, it is easy to formulate a skeptical opinion of the feasibility of mHealth in countries such as Zambia. While it is important to note that mobile phone penetration or mobile network performance alone does not infer broad mHealth readiness, network performance remains a critical factor in preparing for mHealth interventions. Nontechnical factors such as uptake by policy makers and health care practitioners as well as social and cultural acceptance remain integral to the success of mHealth interventions; however, we have chosen to focus on a specific technical indicator [22].

Although mobile phone use has penetrated developing nations to an extent that was unimaginable two decades ago, the implementation of new mHealth interventions in these nations has been met with apprehension from key stakeholders and decision makers [23-26]. Another major source of skepticism is the lack of growth of current projects in the field. Concerns regarding poor connectivity and limitations of accessibility have hampered the success of previous mHealth interventions, and these barriers prevent pilot projects from developing into sustainable, large-scale programs. In Uganda alone, between 2008 and 2009, 23 mHealth projects did not progress beyond pilot testing [12,27]. The failure of these programs to develop beyond preliminary investigations into widespread, integrated mHealth systems increases hesitation, and mobile networks remain an underused health care resource in low- and middle-income countries.

Pilot studies of mHealth interventions often fail to move forward because they cannot overcome the limitations of working with mobile devices in resource-limited settings. Each setting has a unique mobile landscape, and successful strategies are sometimes not generalizable not only from country to country but even within a country or region of interest. Therefore, although the network infrastructure may be in place, even with its clear room for improvement, mHealth pilot studies have not yet succeeded in harnessing that network to produce improved health outcomes. While several factors contribute to overall mHealth readiness, a need remains for tools that can be employed to provide a more complete understanding of the mobile landscape to inform mHealth interventions, particularly in the more remote areas of low- and middle-income countries, where this information is scarce. In this study, we present a mobile phone–based tool, Beacon, to evaluate a setting’s capacity for basic mHealth interventions.

## Methods

### Purpose

We envisioned the Beacon app as a platform to evaluate the infrastructure of a global health setting for readiness to implement mobile health interventions. While there are several existing methods to measure cellular signals, these technologies are often limited by a lack of generalizability, inability to differentiate errors, failure to permanently record any data collected, and requirement for repeated manual intervention to initiate data collection. To rigorously document and assess a region’s mHealth readiness, it is critical for the ideal tool to be broadly accessible, reliable and to have automated data recording capabilities. As such, the software was designed to collect spatiotemporal data on cellular network capabilities and mimic certain necessities, such as data transmission to and from research databases or electronic medical records. In this case, the files that were repeatedly uploaded and downloaded in the software were small camera phone images (3.37 MB and 1.57 MB in size) of malaria rapid diagnostic tests (RDTs); upload and download of such images is a frequent occurrence in our diagnostic work in malaria elimination campaigns. The images were then used repeatedly to mimic RDT image transfer as an example of a potential mHealth app. However, these files could be readily modified to fit an alternative mHealth intervention, including a mock patient record or a video file.

### Software

**Figure 2:**
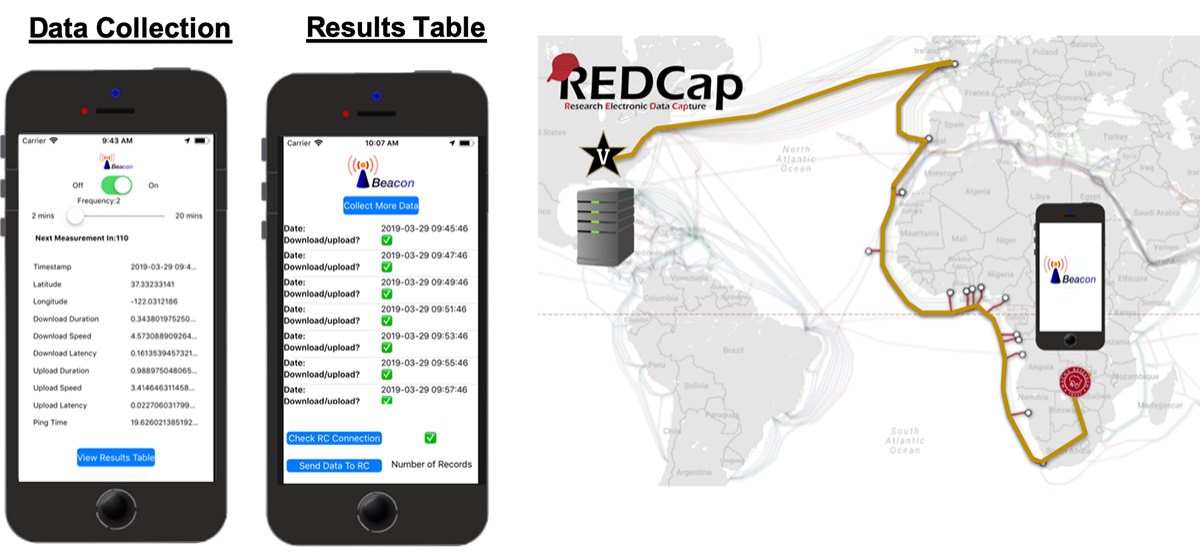
(Left) The two main screens in the Beacon app: the data collection screen and the results table and data transmission screen. (Right) A representative signal trace from the Macha Research Trust to Vanderbilt University, adapted from [13]. Using Beacon, files were downloaded and uploaded to a server at Vanderbilt University. After sufficient data aggregation, data were transferred to REDCap for storage. REDCap: Research Electronic Data Capture.

A custom app was developed in the Swift programming language using the XCode integrated development environment and deployed through the Apple App Store’s beta testing program, TestFlight. The app has two screens: a main screen for data collection and a results screen for data transmission. Collected data are reported as time, latitude, longitude, ping duration, download latency, total download duration, download speed, upload latency, total upload duration, and upload speed (Multimedia Appendix 1). The main view of the app has a simple interface with four components (Figure 2): 1) an on/off switch that allows the user to start and stop data collection, 2) a slider that allows the user to select the frequency of data collection, 3) a table containing the most recent data, and 4) a button to send the user to the Results table, where they can transmit their results to the Research Electronic Data Capture (REDCap) electronic research database [28]. The user can toggle data collection on using the on/off switch, at which point a timer is started with the duration specified by the frequency selected by the user. When the timer expires, the software asynchronously performs the following events using third party libraries (Alamofire v4.7 and PlainPing v0.4): a timestamp is recorded, GPS coordinates are recorded, a ping is sent to a physical server located in the Wright Laboratory at Vanderbilt University, a preloaded image of a rapid diagnostic test is downloaded from the server to the mobile phone, and a different preloaded rapid diagnostic test image is uploaded from the mobile phone to the server. For work in remote regions with unknown connectivity, the geolocation and timestamp are still recorded in the event that an upload, download, or ping fails. All the data describing each interaction between the mobile phone and physical server are entered into a data structure that can be visualized on the results screen. On the results screen, a table displays an entry for each set of collected values. The table row entry shows the timestamp of the data and a check/x symbol depending on if there were any errors in the data entry in that row. At the bottom of the screen, a button (“Check RC Connection”) enables the user to check whether their mobile device was able to connect to REDCap for long-term data storage. If this connection failed, the user is notified, and the user’s data remain in storage for later upload. If a connection can be established, another button (“Send Data to RC”) is enabled; when this button is clicked, the entire data structure describing the interactions of the mobile phone with the VU server is transferred from the app to the web-based REDCap platform. Upon successful data upload to REDCap, the number of data entries uploaded is shown and the data table is cleared.

### Hardware

The software was tested for compatibility on multiple iOS devices, including an iPhone 8 and an iPhone X. The data collection in Macha was performed on multiple iPhone SE (2017) phones, all running iOS 12.1.3. While Apple products are less common globally than they are in the United States, this model has comparable hardware to that in commonly used mobile devices, including the processor, memory, and camera. Therefore, this iPhone is a reasonable device for this testing, with the understanding that the software can readily be ported to Android devices, which are more common in low- and middle-income countries. Data collection was performed on the three primary cellular networks operating in and around Macha, Zambia: Airtel, MTN, and Zamtel. While evaluation of the performance of a particular carrier is an expected use of Beacon in practice, in this work, we randomly assigned each network an alphabetical code to avoid explicitly identifying network performance.

### Data Collection Using the Beacon App

After the timer was initiated in the app, the mobile device was placed in Guided Access mode. This mode allows the user to turn off interactions with the screen or device buttons, allowing the app to collect data uninterrupted by inadvertent screen taps. The app was iteratively and collaboratively refined over the course of 2 weeks before data collection began. Data were collected by three separate users during the course of routine field work in and around the Macha Research Trust between the months of February and August 2019. Users were provided instructions and documentation on how to use the app but were not given any other instructions related to data collection frequencies, locations to visit or to avoid, or how often to upload their results to REDCap. Data were downloaded from REDCap for offline statistical analysis.

## Results

### Screening Mobile Carrier Efficacy Using Beacon

**Figure 3:**
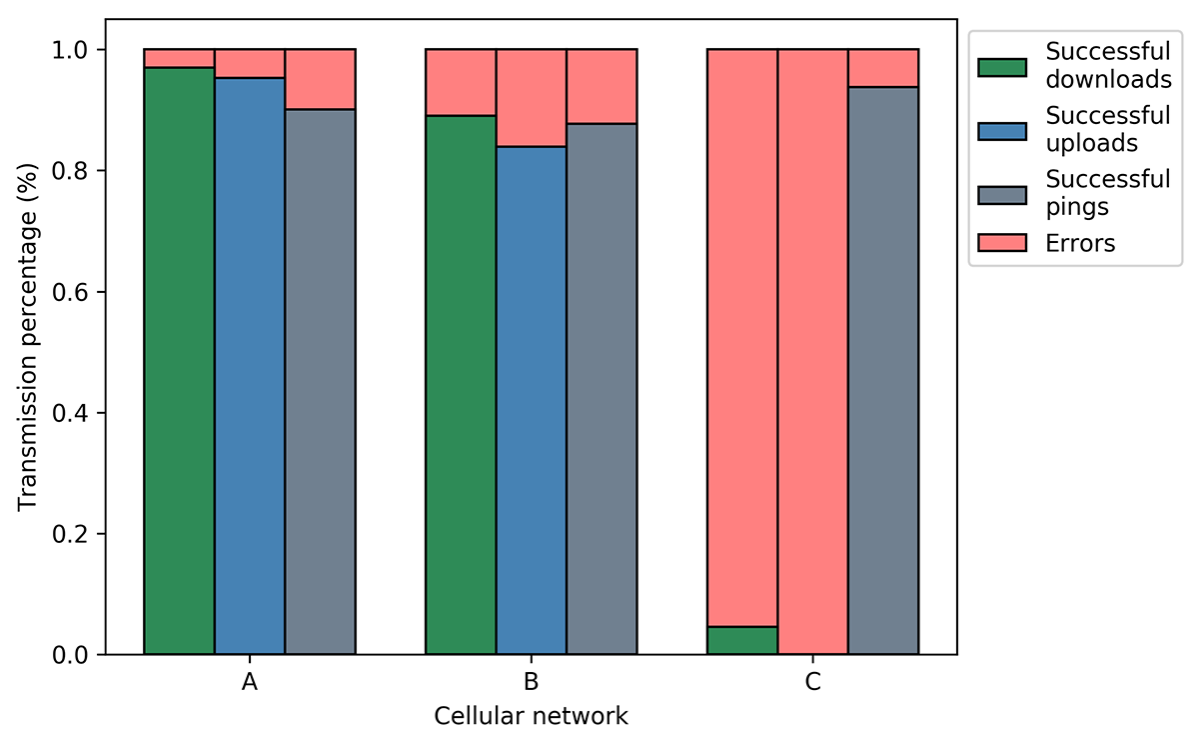
Success and error rates of different modalities of data transfer (download, upload, ping) across three different cellular provider networks.

The Beacon app was used to compare the three primary mobile phone carriers operating in or near Macha, Zambia. Figure 3 shows the results of this comparison, delineated by the transmission success rate of each individual modality: downloads, uploads, and pings. Carriers A and B performed with high success; Carrier A completed 6820/7034 (97.0%) successful downloads and 1431/1608 (89.0%) successful uploads, while Carrier B completed 6701/7034 (95.3%) successful downloads and 1349/1608 (83.9%) successful uploads. However, Carrier C was observed to successfully complete only 62/1373 (4.5%) file downloads and 0/1373 (0.0%) file uploads despite a high rate of successful pings. These results agree with anecdotal evidence reported by Macha residents. The remaining data shown in this report focus on Carrier Network A and on upload duration and download duration as two of the most tangible metrics of cellular network performance.

**Figure 4:**
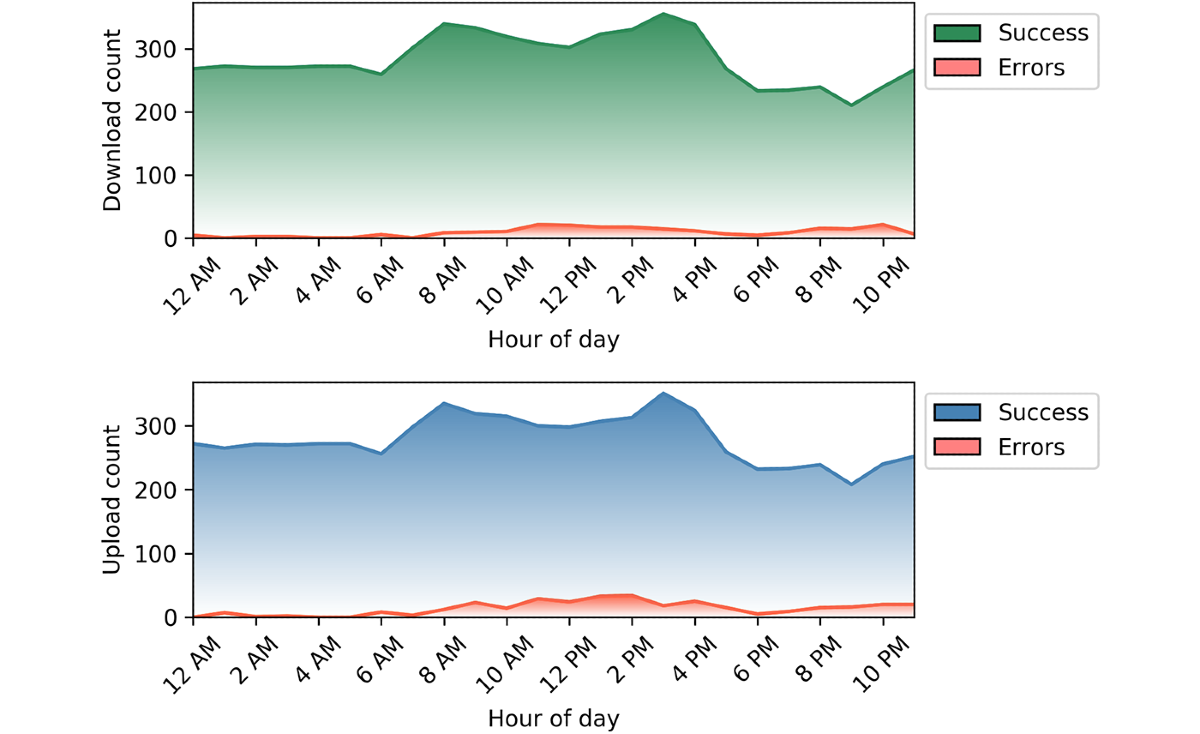
Download (top) and upload (bottom) success and error counts for cellular network A grouped by hour of the day.

Data collection was not fixed at a single defined frequency throughout the entire study, resulting in a nonuniform distribution of record counts based on the day and time of collection (Figure 4). The largest amounts of data were collected at midday and on weekends. As the system was designed, the number of download attempts matched the number of upload attempts (Multimedia Appendix 2). Errors were mostly observed to increase proportionally as the total data count increased; thus, we did not see sampling bias in our results. However, there was slight disagreement between the number of download errors and the number of upload errors in our results, indicating that success of one mode of transmission did not guarantee success of another.

**Figure 5:**
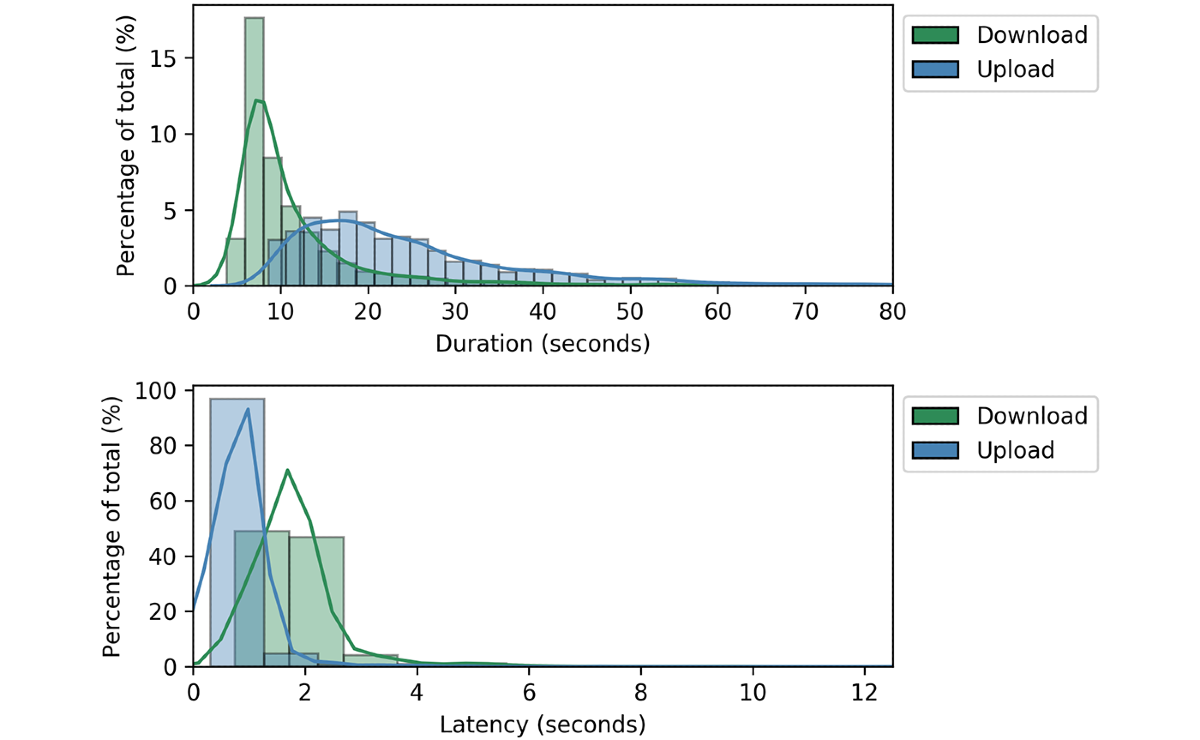
Distributions of upload and download duration (top) and latency (bottom) recorded for Carrier A over the course of Beacon testing. Data outside 1.5 times the interquartile range of the lower and upper quartiles were considered outliers and were removed from the analysis.

The distributions of the upload and download durations recorded for successful data transmissions on Carrier A are shown in Figure 5. On average, downloads occurred faster and in a narrower time range than uploads. To this point, 50% of downloads were completed in under 8.5 seconds and 75% were completed in under 13 seconds, whereas 50% and 75% of uploads were completed in 21 and 30.5 seconds, respectively; the fastest and slowest download times were 3.7 seconds and 21.2 seconds, respectively, while the fastest and slowest upload times were 8.4 and 53.8 seconds, respectively. The average download latency was larger than the average upload latency (1.8 seconds and 0.8 seconds, respectively).

### Spatial Resolution of Cellular Data Transfer Capabilities in Rural Zambia

**Figure 6:**
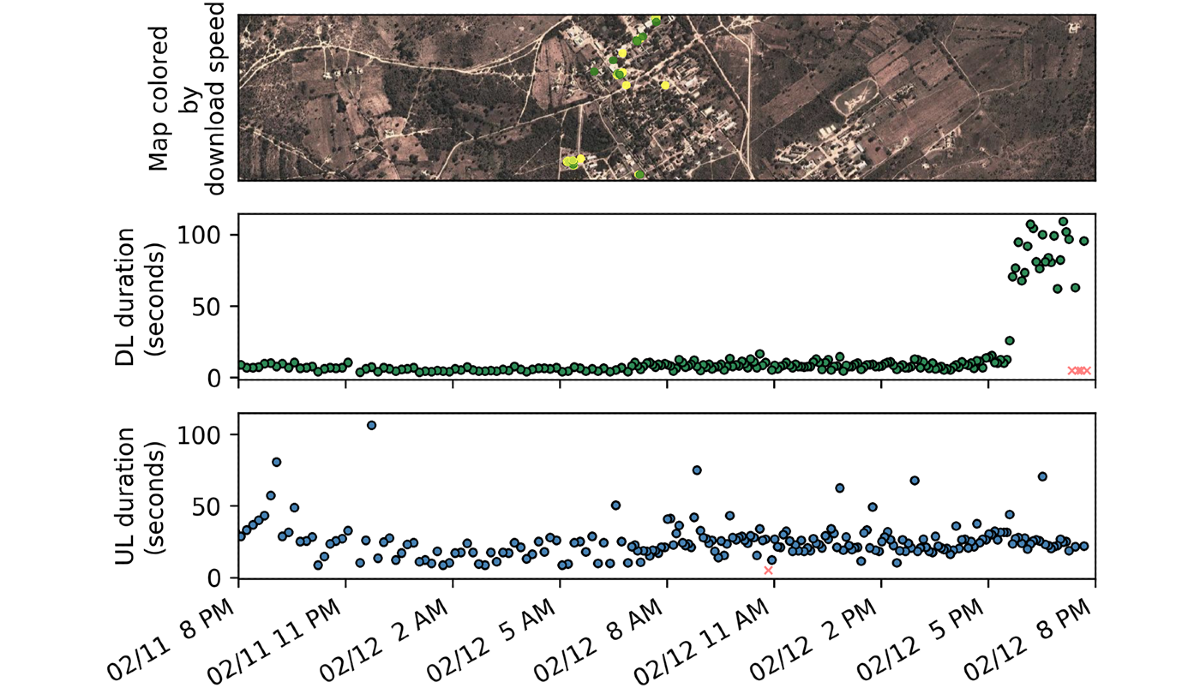
A representative 24-hour period of data collection: (top) download speed mapped by GPS coordinates, where download speed is denoted on a scale from green (high) to yellow (low); (middle) download duration over time; and (bottom) upload duration over time for the same period. Errors are denoted with red x symbols. DL: download; UL: upload

All data collected were tagged with both a GPS location and a timestamp. A single representative day of data collection is shown in Figure 6. As can be seen, the signal strength can be overlaid on a map as a point map with respect to the coordinates from which the data were collected. In and around our study site, the signal strength was found to be high, with most downloads lasting between 4 and 12 seconds. For the data shown, an abrupt increase in upload duration begins just after 5 PM local time. These download durations were more than 1.5 times the upper quartile and are statistical outliers. More variation, including several other outliers, can be observed in the temporal upload duration results; however, the increased variation shown in the representative data matches the overall observed trends shown in Figure 5.

**Figure 7:**
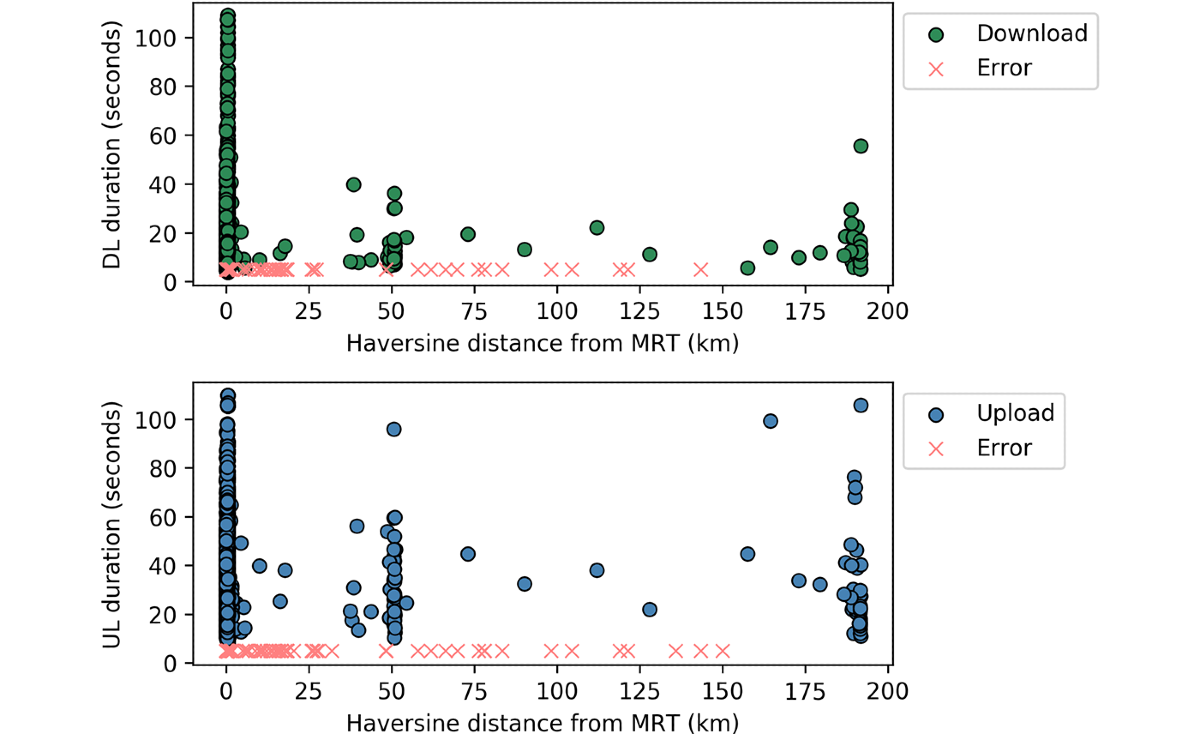
Download (top) and upload (bottom) durations and the errors in each mode as a function of the haversine distance from the Macha Research Trust. DL: download; km: kilometers; MRT: Macha Research Trust; UL: upload.

Our primary study site was located in Macha (a rural village in the Southern Province of Zambia), and the overwhelming majority of our data were collected in and around Macha. However, users were not geographically constrained, and data were collected at other sites, including a larger town (Choma, 50 kilometers away) and a larger city (Livingstone, 190 km away). The durations of successful downloads and uploads are shown as a function of the haversine distance from the Macha Research Trust in Figure 7. While the distance measured from Macha does not have explicit directionality and thus cannot explicitly confirm location by itself (eg, if a user traveled 50 km in the opposite direction, this plot would still show the data points next to Choma), confirmation of the GPS coordinates shows that the overwhelming majority of data points near 50 km and 190 km from the Macha Research Trust were collected in Choma and Livingstone, respectively. Near the Macha Research Trust, the full ranges of download and upload durations were observed; meanwhile, in Choma and Livingstone, the cellular signal was generally more consistent, but outliers were still occasionally observed. In the regions between these cities, higher numbers of errors were reported, which is probably directly related to the distance from the cell towers along the routes traveled and thus more indirectly related to the lack of densely populated areas between cities.

### Temporal Distribution of Cellular Data Transfer Capabilities in Rural Zambia

**Figure 8:**
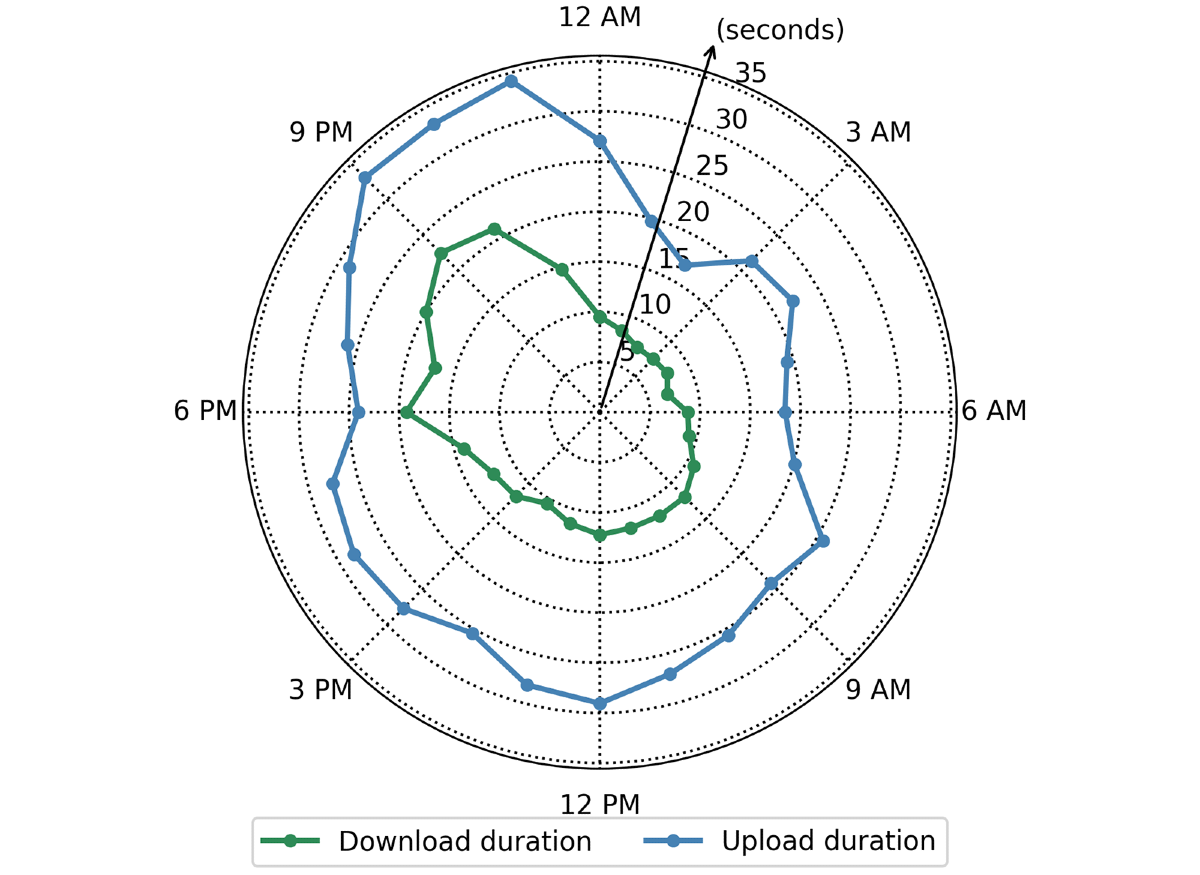
Average download and upload durations for each hour of the day.

When the data are grouped by hour of the day (Figure 8), the download durations are short early in the morning (from 2 AM to 5 AM), followed by an increase from 5 AM to 8 AM. The durations remain roughly constant from 8 AM to 4 PM, with a slight decrease at 2 PM. Download durations experience a significant increase in the evening hours beginning at 5 PM and lasting through 11 PM, until they begin to decrease again (12 AM to 2 AM). A similar but slightly less pronounced and more noisy trend was observed for upload duration. When the data are grouped by day of the week, on average, the download durations are fastest on Monday (10.8 seconds) and slowest on Tuesday (15.5 seconds); on average, the upload durations are fastest on Sunday (21.8 seconds) and slowest on Friday (31.6 seconds; Multimedia Appendix 3). Upload and download latency reflected a similar, although more noisy, trend (Multimedia Appendix 4).

## Discussion

### Principal Findings

In this study, we developed and validated a tool designed to assist readiness evaluation of prospective sites for the implementation of mHealth intervention. In this study, we utilized the Beacon app to examine mobile network performance in and around Macha, Zambia. During this study, we observed several key characteristics of the Macha mobile network, including hourly variations in the network signal and spatial distribution of the signal strength. However, we note that each mHealth site is unique and will have its own variations in network performance. Our results in Macha, Zambia, may be similar to those in other low- and middle-income countries; however, we recommend that each new site be investigated before mHealth interventions are pursued.

The Beacon app enables spatiotemporal mapping of two of the most practical measures of network performance: upload speed and download speed. We acknowledge that network performance alone does not confer mHealth readiness; however, understanding the digital landscape of a potential mHealth intervention site is a critical step in assessing the feasibility of future mHealth studies. Data collection in Beacon is fully automated, and data are stored remotely in an easily accessible research database. The Beacon app can detect errors in either upload or download attempts and log them with the same geographic and temporal precision provided for successful attempts. The app can be readily tailored based on the needs of researchers to customize data collection and aggregation to fit the intervention of interest.

Beacon was tested in Macha, Zambia, and was critical in developing a more well-rounded understanding of the mobile landscape around this rural village. In this study, we were able to identify the best-performing cellular network for use in potential mHealth interventions and, in this case, validate the anecdotal experiences of Macha residents. Although we observed significant differences between the available networks, variations in network performance are to be expected in any environment. Network performance is a function of many elements, including but not limited to the number and locations of available cell towers, obstructions between towers (eg, buildings), cell tower hardware, mobile unit hardware, and current network load at a given time [29]. Many of these factors can be determined before or during testing and, combined with Beacon verification, can further influence decisions reached by mHealth researchers.

In addition to a comparative evaluation of multiple networks, Beacon was used to quantitatively measure upload and download metrics around the test site. From anecdotal experience, download speed in Macha was observed to be faster than upload speed, and this was confirmed during data collection. In most scenarios, this is to be expected due to the asymmetric design and operation of cellular networks [30]. However, despite the increased speed of downloads compared to uploads, download latency was observed to be higher than upload latency. This result is counterintuitive in light of the observed trends in total download/upload duration but may be related to the asymmetry of network configurations. However, overall latency in both directions was low and did not exceed the acceptable latency for single-item transfer, which is a positive finding given the physical distance between the mobile phone (Macha, Zambia) and the home server (Vanderbilt University, Nashville, TN) [31].

Even the simple raw data from Beacon can be transformed into more information-dense results; for instance, data can be grouped temporally (by time of day and day of week). We saw no discernible trend for upload or download speed based on the day of the week; however, the hourly data did appear to correlate with times of high human activity in the catchment area. Between approximately midnight and 6 AM, upload and download durations were at their lowest level, and they began to increase between 6 AM and 8 AM. This is presumably related to many people waking and beginning to use their mobile devices. During working hours, this moderate activity remained and continued until approximately 5 PM, when mobile phone users leave work and more freely engage with their mobile devices. These data are useful for mHealth interventions, as they provide both an optimal window for large-volume data transfer or other network activities and a proxy for when users are on their mobile devices. Based on this information, an mHealth implementation team may decide to schedule large data backups in the early hours of the morning, when the burden on the local network is low and it would thus be more capable of high-capacity use; they may also decide that lightweight SMS text message interventions will be more successful at times of higher network activity.

The spatial resolution of Beacon is also a strength for mHealth researchers who are planning interventions. Plots of signal strength mapped over topographical or satellite images can help visualize the digital landscape and can be useful to denote strength based on distance from landmarks such as cell towers, hospitals, and airports. This, in turn, can help inform researchers interested in implementing both localized and broadly spaced interventions. Further collaboration between mHealth researchers, local municipalities, and internet service providers could enable the use of these data to inform strategic infrastructure improvements.

As mentioned previously, users were not provided any instructions regarding the frequency of data collection. Among the strengths of the Beacon app are that it can be easily powered on and off and that the frequency of data collection can be adjusted over a broad range. While high-frequency data collection provides more granular temporal and spatial data, it does come at a higher overall cost. With a small file size and at the minimum collection rate (collection every 20 minutes), Beacon costs less than 30 kwacha per week (approximately US $2). These operating costs would be expected to increase proportionally with higher frequency or larger download/upload file size; however, high spatiotemporal data can still be collected for <US $100 per month. This potential increase in cost experienced by varying these parameters may more closely approximate the actual intervention at a site; therefore, it may prove to be worthwhile to mHealth investigators when using Beacon-based surveillance. Although we did not observe sampling bias during this initial test, we do recognize the potential for variable sampling frequency to introduce bias into a preliminary study. For instance, if rolling outages were to occur during a certain time of day in a study location and collection frequency was increased during this time, further analysis of sampling practices might be required to account for the higher volume of signal errors. This requires diligent monitoring and analysis or, as a more straightforward solution, the implementation of a strict data collection frequency that reflects the needs of the planned mHealth intervention.

### Conclusions

Health care remains a considerable challenge in many parts of the world, particularly resource-limited countries. Even without continuous connectivity, mHealth interventions have genuine potential to improve global health outcomes. Although many countries have high mobile phone penetration, not all locations are capable of supporting mHealth. The lack of infrastructure and high costs may be prohibitive; however, proper planning and tailoring of these interventions based on the infrastructure available greatly increases the likelihood of success. The small cost of piloting a short signal mapping study, such as this one, would be negligible compared to the cost of blind implementation of an intervention that is destined to fail. Beacon represents a valuable mHealth tool that can help determine the readiness of a site for mHealth interventions. By combining temporal and spatial tags with metrics of network performance, researchers can better understand the digital landscape around their potential intervention site.

## Appendix

Multimedia Appendix 1 The Beacon application user interface and workflow.

Multimedia Appendix 2 Download (top) and upload (bottom) duration grouped by hour of the day (left) and day of the week (right).

Multimedia Appendix 3 Number of successful and unsuccessful download (top) and upload (bottom) data points collected, grouped by day of the week.

Multimedia Appendix 4 Download (top) and upload (bottom) latency grouped by hour of the day (left) and day of the week (right).

## Abbreviations

mHealth: mobile health
NRI: Network Readiness Index
RDT: rapid diagnostic test
REDCap: Research Electronic Data Capture
