# Supplementary material for: Evaluating Network Readiness for mHealth Interventions Using the Beacon Mobile Phone App: Application Development and Validation Study
Source: JMIR Mhealth Uhealth. 2020 Jul 28;8(7):e18413. doi: 10.2196/18413 (PMC7420690; doi:10.2196/18413)
Supplement: Multimedia Appendix 1 [file mhealth_v8i7e18413_app1.docx]

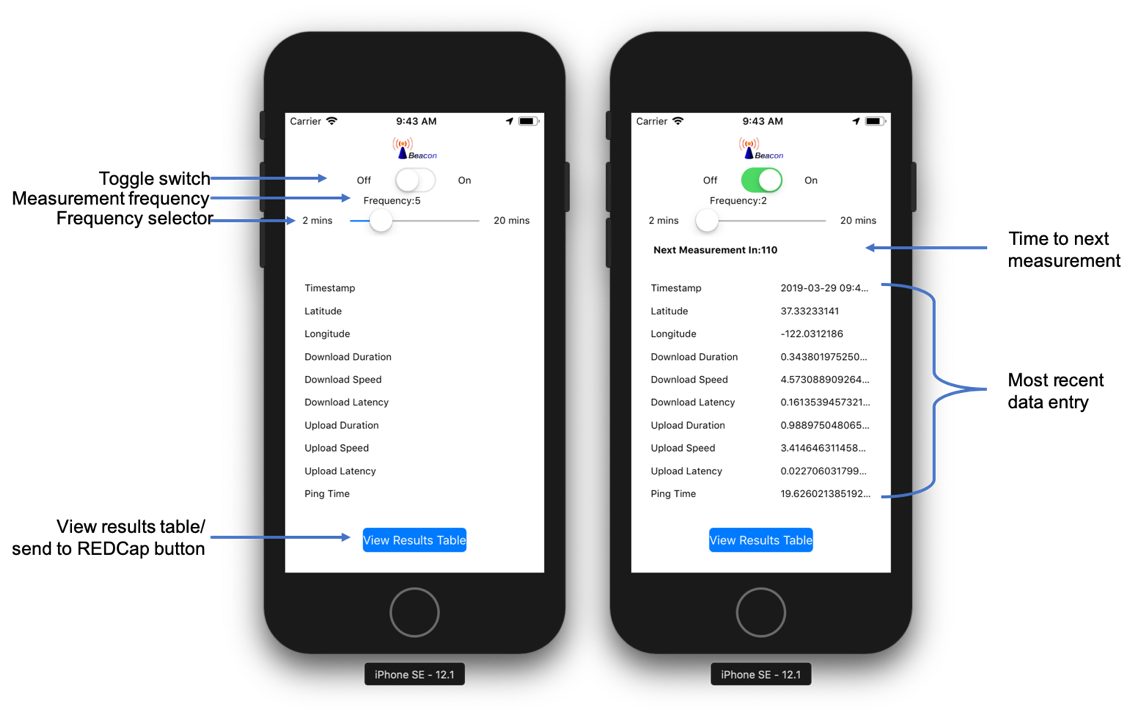

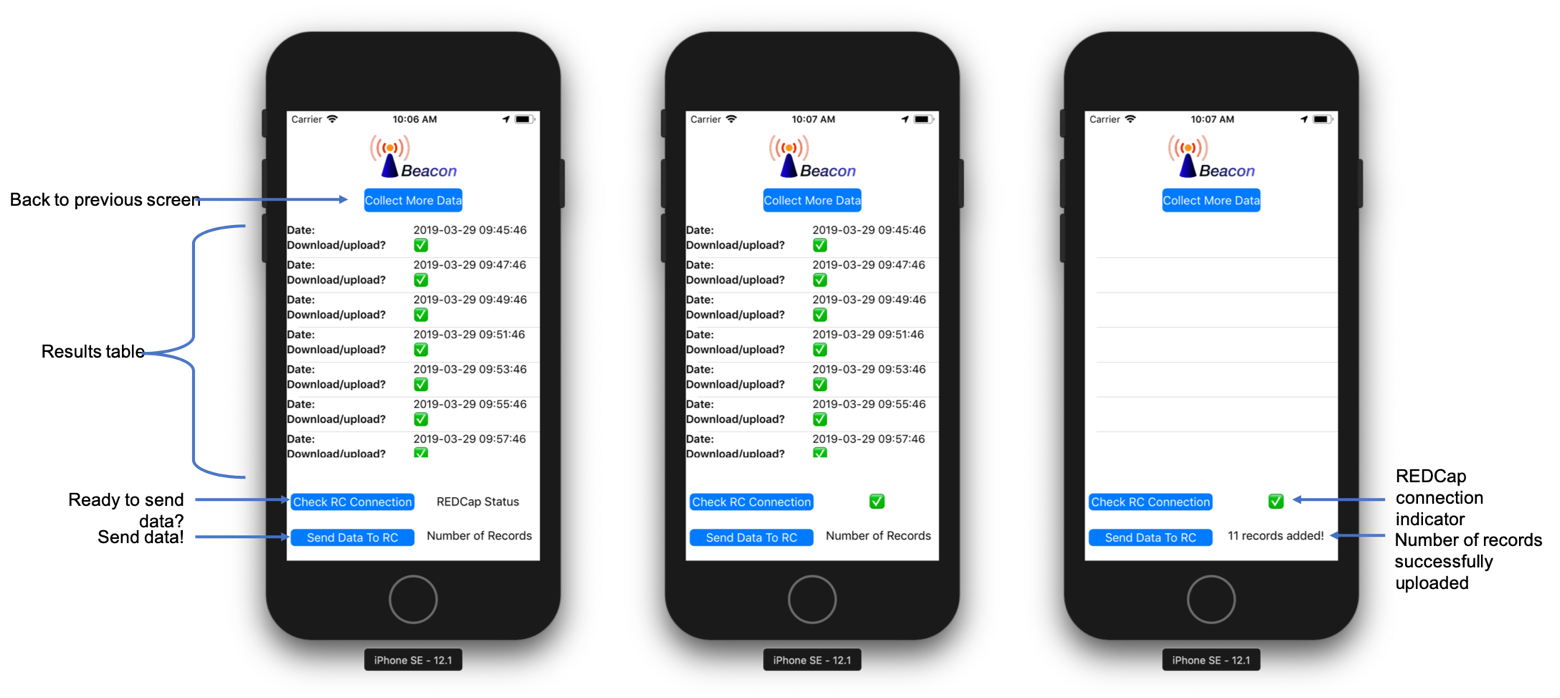


**The Beacon application interface:** Upon opening the app (top left), users have the option of toggling data collection on or off. Users can also change the frequency of data collection using the slide bar. The frequency counter above the bar reflects the current data collection frequency. When data collection begins (top right), each data field (e.g. timestamp, location, download and upload measurements) will populate with the most recent data points and a countdown timer will be initiated to display time to next measurement. To push data to the REDCap database, users must push the blue “View Results Table” button. This button will direct them to the data table screen (bottom).

Once users have navigated to the results table screen, they have the opportunity to return to data collection via the blue “Collect More Data” button at the top of the screen. If users are finished with a collection session, they can visually scan the individual data points for success or error (indicated by green checks or red x’s) within the data table at the center of the screen (bottom, left). To upload data to REDCap, users then press the blue “Check RC Connection” button. This button initiates contact with the REDCap server. A green check box will appear next to the button when a successful connection is established (bottom center). The user then pushes the blue “Send Data to RC” button to upload the full data set to REDCap. When the upload is complete, all data is removed from the table. This indicates that the app is ready to collect again.
