# Supplementary figures and images for: Evaluating Network Readiness for mHealth Interventions Using the Beacon Mobile Phone App: Application Development and Validation Study
Source: JMIR Mhealth Uhealth. 2020 Jul 28;8(7):e18413. doi: 10.2196/18413 (PMC7420690; doi:10.2196/18413)

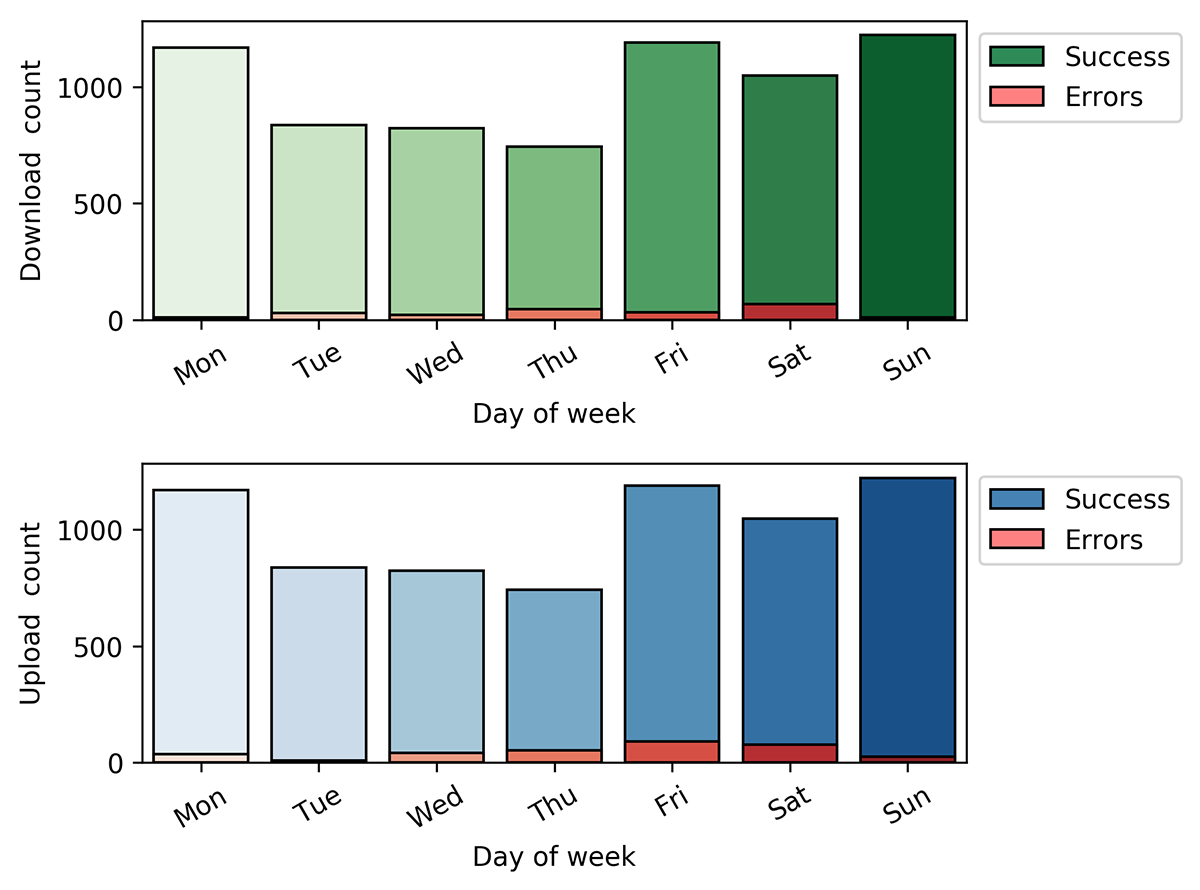

Supplement: Multimedia Appendix 2 [file mhealth_v8i7e18413_app2.png]

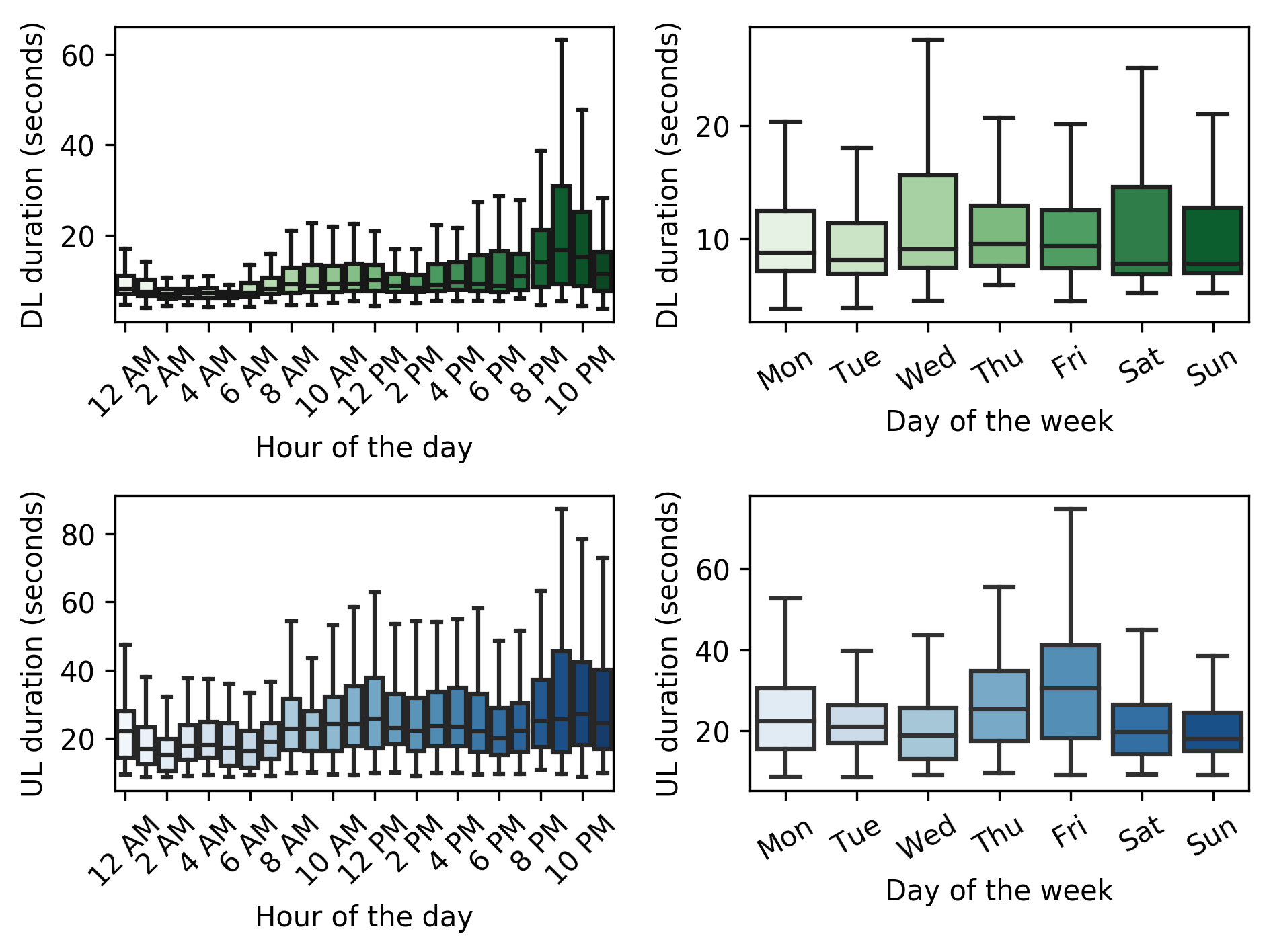

Supplement: Multimedia Appendix 3 [file mhealth_v8i7e18413_app3.png]

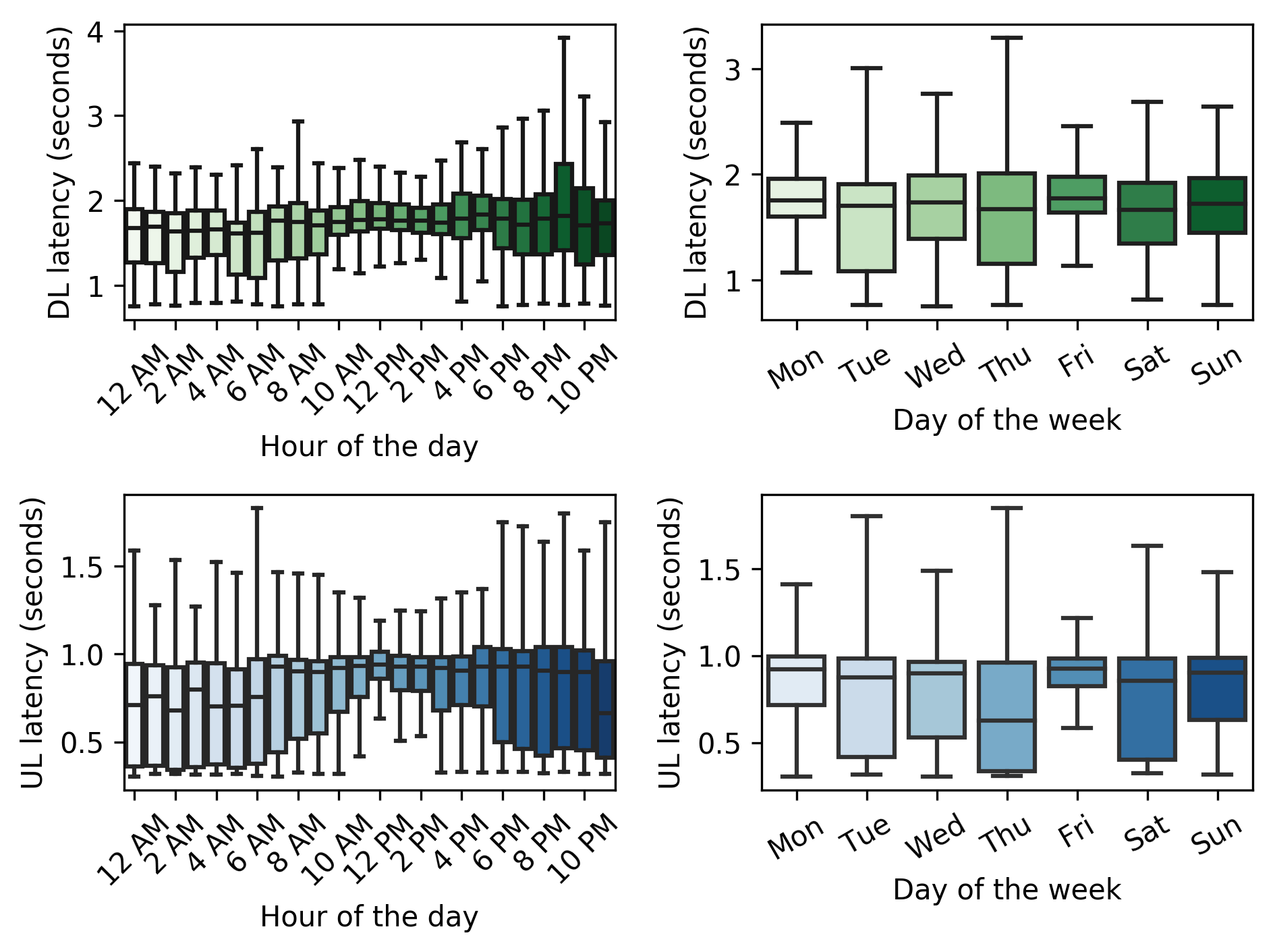

Supplement: Multimedia Appendix 4 [file mhealth_v8i7e18413_app4.png]
